# The Essential Role of IL-17 as the Pathogenetic Link between Psoriasis and Metabolic-Associated Fatty Liver Disease

**DOI:** 10.3390/life13020419

**Published:** 2023-02-02

**Authors:** Antonio Olveira, Salvador Augustin, Salvador Benlloch, Javier Ampuero, Jorge Alonso Suárez-Pérez, Susana Armesto, Eva Vilarrasa, Isabel Belinchón-Romero, Pedro Herranz, Javier Crespo, Francisco Guimerá, Lara Gómez-Labrador, Víctor Martín, José Manuel Carrascosa

**Affiliations:** 1 Department of Digestive Diseases, La Paz University Hospital, 28046 Madrid, Spain; 2 Liver Unit, Vall d’Hebron Hospital Universitari, Vall d’Hebron Institut de Recerca (VHIR), Vall d’Hebron Barcelona Hospital Campus, Universitat Autònoma de Barcelona, 08193 Barcelona, Spain; 3 Department of Digestive Diseases, Arnau de Vilanova Hospital, Centro Biomédico en Red de Enfermedades Hepáticas y Digestivas (CIBERehd), 46015 Valencia, Spain; 4 Department of Digestive Diseases, Virgen del Rocío University Hospital, Lab 213, Institute of Biomedicine of Sevilla (IBIS), Department of Medicine, University of Sevilla, Centro Biomédico en Red de Enfermedades Hepáticas y Digestivas (CIBERehd), 41004 Sevilla, Spain; 5 Department of Dermatology, Virgen de la Victoria University Hospital, 29010 Málaga, Spain; 6 Department of Dermatology, Marqués de Valdecilla University Hospital, 39008 Santander, Spain; 7 Department of Dermatology, Santa Creu i Sant Pau Hospital, Universitat Autònoma de Barcelona, 08193 Barcelona, Spain; 8 Dermatology Department, Alicante University General Hospital, Institute for Health and Biomedical Research (ISABIAL), Miguel Hernández University of Elche, 03202 Alicante, Spain; 9 Department of Dermatology, La Paz University Hospital, 28046 Madrid, Spain; 10 Gastroenterology and Hepatology Department, Marqués de Valdecilla University Hospital, IDIVAL, School Medicine, University of Cantabria, 39005 Santander, Spain; 11 Dermatology and Pathology Department, Canarias University Hospital, 38320 La Laguna, Spain; 12 Immunology Franchise, Novartis Farmacéutica S.A., 28033 Madrid, Spain; 13 Department of Dermatology, Germans Trias i Pujol University Hospital, Universitat Autònoma de Barcelona, IGTP, 08193 Badalona, Spain

**Keywords:** psoriasis, metabolic-associated fatty liver disease, MAFLD, IL-17, IL-17A

## Abstract

Interleukin 17 (IL-17) is an effector cytokine that plays a key role in the pathogenesis of both psoriasis and metabolic-associated fatty liver disease (MAFLD), a condition that is more prevalent and severe in patients with psoriasis. In liver inflammation, IL-17 is mainly produced by CD4+ T (TH17) and CD8+ T cells (Tc17), although numerous other cells (macrophages, natural killer cells, neutrophils and Tγδ cells) also contribute to the production of IL-17. In hepatocytes, IL-17 mediates systemic inflammation and the recruitment of inflammatory cells to the liver, and it is also implicated in the development of fibrosis and insulin resistance. IL-17 levels have been correlated with progression from MAFLD to steatohepatitis, cirrhosis, and even hepatocellular carcinoma. Clinical trials have shown that inhibiting IL-17A in patients with psoriasis could potentially contribute to the improvement of metabolic and liver parameters. A better understanding of the key factors involved in the pathogenesis of these chronic inflammatory processes could potentially lead to more efficient treatment for both psoriasis and MAFLD, and help to develop holistic strategies to improve the management of these patients.

## 1. Introduction

Psoriasis is one of the most common chronic inflammatory diseases in Western countries, with a prevalence rate ranging from 0.51–11.43% in the adult population worldwide, and 2.69% in Spain [1,2,3]. Psoriasis is frequently associated with other inflammatory diseases, such as psoriatic arthritis, obesity, metabolic syndrome (MetS), cardiovascular comorbidities, neurological disorders and inflammatory bowel disease, among other conditions [2,3,4].

Although the precise pathogenic biological mechanisms underlying the association between psoriasis and these other inflammatory conditions are not fully understood, it has been postulated that common inflammatory pathways, cellular mediators, genetic susceptibility and certain risk factors could be involved in the development and establishment of coexisting inflammatory diseases [2]. Metabolic-associated fatty liver disease (MAFLD), formerly referred to as non-alcoholic fatty liver disease (NAFLD), affects up to 25% of the world population [2] and is commonly linked with psoriasis. MAFLD is the most common cause of liver disease [5] and the second leading cause of liver transplantation in the United States [6]. In Spain, estimates suggest that up to 25.8% of the general population may have MAFLD. The incidence of this disease continues to rise, following the growing prevalence of obesity and diabetes, which could eventually lead to a major public health crisis if these trends continue in the future [7,8]. Despite this global concern, there are currently no pharmacological treatments for MAFLD.

MAFLD is characterized by fat deposition in more than 5% of the hepatocytes, evidenced by imaging scans or histological assessment, in the absence of secondary causes of steatosis, such as significant alcohol consumption, steatogenic drugs or hereditary disorders [9]. In most cases, MAFLD is associated with metabolic risk factors, such as diabetes, obesity and dyslipidemia, all of which metabolically stress the liver, leading to insulin resistance and inflammation. Hepatic steatosis is a quite stable stage of liver disease characterized by the accumulation of intracellular fat in hepatocytes. The sources of this accumulated fat include dietary fat, liver production, and release from insulin-resistant adipose tissue. However, in a significant percentage of cases, MAFLD can evolve into non-alcoholic steatohepatitis (NASH). Several studies have situated the prevalence of NASH among MAFLD patients at 29.9%, with an increasing tendency in recent years, up until a prevalence of 59.1% [10]. The progression from MAFLD to NASH occurs when fat infiltration causes cell damage, inflammatory changes and, sometimes, fibrosis (a healing process that occurs when collagen fibers replace dead cells). These fibrotic changes lead to different degrees of liver fibrosis. The parenchyma structure is then progressively substituted by fibrous septa and regenerative nodules. If fibrosis persists unopposed, the damage to the parenchymal structure causes a progressive loss of liver function and ultimately leads to cirrhosis. Patients with advanced liver disease have a high risk of developing hepatocellular carcinoma (HCC) and liver failure, which may require liver transplantation, which remains the leading causes of death in this population [8,11,12,13,14]. Estimates suggest that 10–15% of patients with MAFLD have liver fibrosis. Furthermore, HCC affects approximately 14% of patients with MAFLD and liver fibrosis [15]. However, one of the most significant disadvantages related to MAFLD is that, once it is established, it contributes to insulin resistance and cardio-metabolic risk factors, especially in non-obese, non-diabetic individuals [16].

The prevalence of MAFLD in patients with psoriasis has been reported to be double the rate observed in the general population, and its prevalence could reach as high as 65% among this patient population [17,18]. In fact, MAFLD has been shown to occur 1.5 to 3 times more frequently in patients with psoriasis than in the general population [19,20,21]. Moreover, patients with psoriasis tend to have more severe liver disease, which is directly correlated with the severity of psoriasis [22]. A recent Spanish study showed that 42.3% of the psoriatic patients presented MAFLD, and that patients with MAFLD had a higher absolute psoriasis area severity index (PASI), body surface area (BSA) and physician’s global assessment (PGA) than those with psoriasis alone [20]. Another recent study in Spain found that 52.1% of psoriatic patients had concomitant MAFLD, and 14% of these patients had a high risk of developing liver fibrosis [21]. Patients with psoriasis have a higher risk of death due to liver failure, which correlates with psoriasis severity [23]. This high mortality risk is generally associated with these patients’ high prevalence of MAFLD. In this setting, the use of systemic hepatotoxic treatments, such as methotrexate (MTX), can contribute to the development of liver fibrosis [23]. A study conducted in Spain in patients (n = 497) treated with MTX found that 64.1% had hepatic steatosis. Moreover, based on the NAFLD fibrosis score and the Fibrosis-4 Index for Liver Fibrosis score, 37.2%, and 26.2%, respectively, were considered to have an intermediate-to-high risk of developing liver fibrosis, which was also associated with a longer duration of treatment [24].

MAFLD is frequently considered to be the hepatic manifestation of MetS, although it can also precede MetS, type 2 diabetes mellitus (DM2) and arterial hypertension [25,26]. Additionally, it is known that, after the age of 40 years, patients with psoriasis have a higher prevalence of MetS and an increased risk for each of its components compared to controls [27]. Chronic inflammation and insulin resistance appear to be the main pathogenic links between psoriasis and MAFLD [18]. Many studies have evaluated the role of interleukin 17 (IL-17) as a key mediator in the pathogenesis of these and other inflammatory conditions [28,29].

In this context, the present study aimed to review the literature on the role of IL-17 in systemic inflammation in MAFLD and psoriasis to elucidate the pathogenic link between these two diseases.

## 2. Materials and Methods

This review was performed by a 12-member multidisciplinary work group comprising dermatologists, gastroenterologists, internists and hepatologists, specialized in psoriasis and MAFLD. This expert group drew up a list of points to be reviewed and updated to summarize recent and relevant findings. For this, the work group conducted several literature searches using the PubMed database in 2020. The following Mesh terms were searched: “psoriasis” AND “NAFLD”, “psoriasis” AND “NASH”, “IL-17” AND “NAFLD”, “IL-17” AND “NASH”, “IL-17” AND “liver inflammation”, «IL-17» AND «liver fibrosis», «TH17» AND «NAFLD», and «TH17» AND «NASH». Subsequently, the expert group discussed and summarized the key points resulting from this literature review based on the findings of these searches.

## 3. Results

### 3.1. Role of IL-17 in Systemic Inflammation

IL-17 is a central player in the physiological immune response against extracellular bacteria and fungi [29]. However, it also contributes to the pathogenesis of various inflammatory pathologies [28,29,30]. It is synthesized mainly by T helper 17 (TH17) lymphocytes and by other cell types, such as CD8 lymphocytes and other cells of innate immunity, such as natural killer (NK), natural killer T (NKT), Tγδ and ILC3 lymphocytes [29]. The presence of IL-17 is especially important in epithelial cells that are in contact with the outer environment, such as the skin, respiratory tract, oral cavity, gastrointestinal tract and the vagina [30].

The liver is also one of the main producers of IL-17, and the IL-17 receptor is widely expressed in liver cells, such as hepatocytes, sinusoidal cells, biliary cells and stellate cells [31,32]. The activation of the IL-17 pathway has been shown to be a key mediator in the hepatic inflammatory response [33]. During this response, NK, NKT and TH17 cells increase their production of IL-17. Furthermore, these cells are involved in fatty liver injury progression. Similarly, obesity may also contribute to the pathogenesis of fatty liver disease, by increasing the levels of TH17 cells and IL-17 production, which are increased in fatty liver disease [34]. In turn, these high levels of IL-17 induced by NK, NKT and TH17 cells activate the IL-17 receptor in a subset of liver cells, mainly hepatocytes, Kuppfer cells and hepatic stellate cells. By doing so, the IL-17-mediated activation of these cells leads to further pro-inflammatory cytokine and chemokine production, neutrophil recruitment, reactive oxygen species production and increased collagen deposition, which are processes known to mediate MAFLD progression [33].

Notably, overexpression of the IL-17 axis has been associated with a variety of diseases affecting the liver, such as hepatitis B and C, alcohol, primary biliary cholangitis, acute rejection in liver transplant, hepatocellular carcinoma, autoimmune hepatitis and primary sclerosing cholangitis [29]. Furthermore, mouse models have shown that the blockade of IL-17 protects the liver from injury, while its administration increases liver damage [35,36]. For these reasons, the blockade of the IL-17 axis has been proposed as a future therapeutic target that could prove beneficial, although its potential effectiveness requires further investigation [29].

IL-17 stimulates the production of numerous chemokines, such as IL-8, chemokine (C-X-C motif) ligand (CXCL) 1, CXCL2, CXCL5, chemokine (C-C motif) ligand (CCL) 2, CCL7, CCL20; cytokines, such as TNF, IL-6, IL-1β, granulocyte colony-stimulating factor (G-CSF), granulocyte-macrophage colony-stimulating factor (GM-CSF); and other proteins involved in the inflammatory response [28,29]. By itself, IL-17 is not a potent inducer of inflammation. Instead, IL-17’s effects are due to its ability to trigger the recruitment of immune cells by inducing expression of these chemokines and receptors, as well as from synergistic action with other cytokines, such as TNF, IL-1β, IFNγ, GM-CSF and IL-22, thus activating a pro-inflammatory cascade [29]. Although IL-17 is an essential protective cytokine against certain pathogens, excessive activation of this pathway can contribute to a chronic inflammatory state [37], thereby playing a fundamental role in the pathogenesis of many of inflammatory conditions [29].

**Figure 1:**
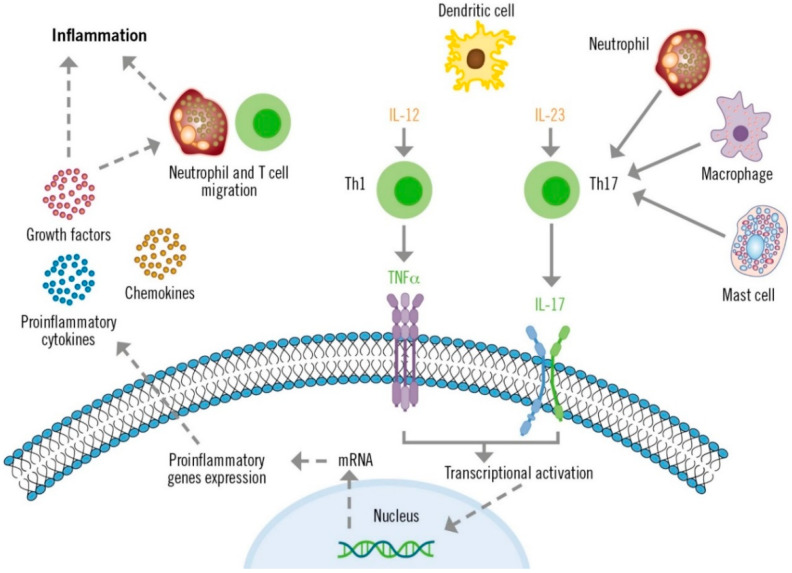
Role of IL-17 in the pathogenesis of psoriasis.

The IL-23/TH17 axis plays a crucial role in the pathogenesis of psoriasis (Figure 1) [29,38]. Dendritic cells produce high levels of IL-23, which stimulate the differentiation and activation of TH17 lymphocytes. Consequently, TH17 cells produce large quantities of IL-17 and other cytokines that directly affect epidermal keratinocytes and other skin cells. These cytokines and TNF act as transcriptional activators of specific genes in keratinocytes. These effects, along with the autoantigenic stimulation response of T lymphocytes, create inflammatory loops that perpetuate lymphocyte activation and the psoriasis phenotype [38]. Additionally, the IL-23-independent IL-17 production by innate immune system cells has also been described in patients with psoriasis who present with plaques refractory to the IL-12/23 inhibitor ustekinumab [39].

### 3.2. IL-17 as the Key Effector Cytokine Link between Psoriasis and MAFLD

Low-grade chronic systemic inflammation appears to be the main link between psoriasis and MAFLD [40,41]. Psoriasis-associated systemic inflammation has also been shown to promote inflammation in adipose tissue [41]. Obesity and increased fatty body tissue contribute to this inflammatory process, in which the production and release of adipokines involved in energy balance (resistin, leptin, visfatin), non-esterified fatty acids and pro-inflammatory cytokines, such as IL-6 and tumor necrosis factor-alpha (TNF-α), are directly related to the development of insulin resistance and MAFLD [20,42]. Simultaneously, there is also a decrease in the concentration of anti-inflammatory adipokines, such as adiponectin, which increase insulin sensitivity in the skin [43]. Ultimately, this disequilibrium leads to liver disease. In turn, the presence of liver disease is believed to increase the severity of psoriasis through a common cascade of cytokines, adipokines and other inflammatory mediators [17], causing a pro-inflammatory feedback loop between the skin, liver and adipose tissue, thus maintaining a perpetual state of systemic inflammation [22].

**Figure 2:**
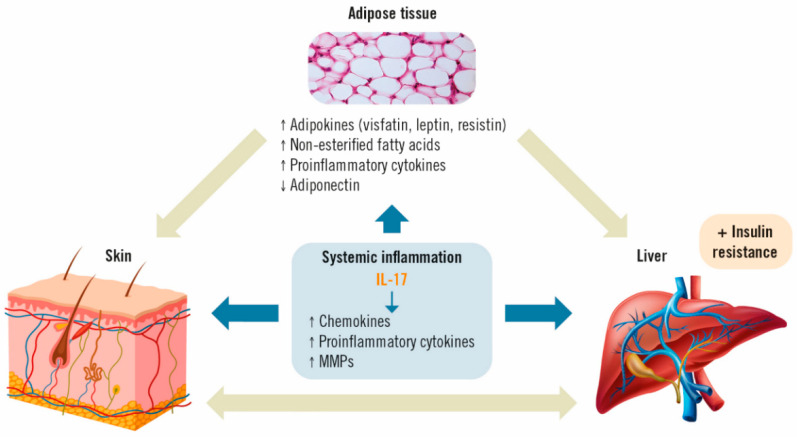
Effects of IL-17 on adipose tissue, psoriasis and MAFLD [35,36]. IL: interleukin; MMP: matrix metalloproteinases.

In this setting, IL-17 acts on different cell types in the skin (dendritic cells, keratinocytes, endothelial cells and fibroblasts) and the liver (stellate cells, hepatocytes, biliary epithelial cells and Kupffer cells). These cells will, in turn, secrete more pro-inflammatory cytokines, such as IL-6, chemokines (IL-8, CCL-20) and other proteins (matrix metalloproteinases; MMP) involved in the recruitment of more inflammatory cells, hence, further perpetuating inflammation (Figure 2) [32,44].

It has also recently been suggested that bacterial translocation from the gut and skin of patients with psoriasis is associated with MAFLD and a higher estimated inflammatory response, as bacterial translocation is more frequent in patients with, versus without, MAFLD [45]. Bacterial translocation may be of interest, since one of the factors in the development and progression of MAFLD is gut permeability, which may be mediated by the microbiome [20]. Moreover, in vitro experiments involving naïve CD4 T cells from healthy and MAFLD subjects showed that endotoxins do not directly act on naïve CD4 T cells, but instead require the presence of antigen-presenting cells to upregulate IL-17. The toll-like receptor 4 (TLR4) is the receptor for endotoxin. Inhibition of TLR4 in macrophages, but not in naïve CD4 T cells, could impair endotoxin-mediated IL-17 upregulation. However, in samples from patients with NASH, endotoxin at high levels increased directly, but minimally, IL-17 production. These data demonstrate that endotoxin promotes TH17 bias in NASH patients [46].

Although the directionality of the association between MAFLD and psoriasis has not been clearly determined, the excess production of pro-inflammatory cytokines produced by lymphocytes and keratinocytes present in psoriatic skin (e.g., IL-6, TNF-α, IL-17) are believed to mediate insulin resistance [42]. The development of insulin resistance is believed to be the first step towards the accumulation of lipids in the liver, with the subsequent activation of downstream cascades leading to lipotoxicity, oxidative damage and apoptosis, finally leading to steatohepatitis and fibrosis [47].

However, several facts suggest a possible bi-directional relationship between psoriasis and MAFLD through the activation of pro-inflammatory pathways linking expanded visceral adipose tissue, steatotic liver and psoriatic skin. These signals pass between these three organs, configuring an hepato-dermal axis [42]. Moreover, these processes, that involve chronic mild systemic inflammation and insulin resistance, are also thought to mediate the association between the severity of psoriasis and MAFLD [48].

In these relationships that configure the hepato-dermal axis, one direction is represented by circulating pro-inflammatory cytokines derived from psoriatic skin, such as TNF-α and IL-17, which produce systemic effects and, upon reaching the liver, could impact liver inflammation by inducing insulin resistance and subsequent metabolic changes, which lead to the development of fatty liver disease. Furthermore, several cytokines, including TNF-α, IL-1, IL-2, IL-6 and IL-17, are known to influence glucose metabolism and insulin sensitivity in hepatocytes and adipocytes, leading to uncontrolled lipolysis and increased hepatic free fatty acid deposition [49]. Conversely, the second direction of the hepato-dermal axis is represented by pro-inflammatory mediators stemming from hepatic inflammation, which could contribute to the onset or exacerbation of cutaneous inflammation in psoriasis. Pro-inflammatory immune modulators released by adipose tissue and the liver are involved in promoting hepatic fibrogenesis in MAFLD, as well as psoriasis pathogenesis [50]. For example, TNF-α is involved in psoriasis inflammation and has been shown to be an independent predictor of hepatic fibrogenesis and disease progression [51]. IL-17, which plays a central role in psoriasis pathogenesis, can induce the activation of hepatic stellate cells and subsequent collagen production. This cell activation is mediated by IL-17, which then facilitates the progression from simple liver steatosis to steatohepatitis [29,43]. Lastly, MAFLD could affect psoriasis severity through the release of inflammatory mediators from the hepatocyte, namely, reactive oxygen species, C-reactive protein and IL-6 [49].

### 3.3. Role of IL-17 in MAFLD

In the development and maintenance of MAFLD, multiple stimuli occurring in the intestine and adipose tissue promote the development of liver inflammation [52]. The low-grade chronic inflammation that is characteristic of steatosis plays a central role in the development of NASH and its progression to fibrosis [12,52].

**Figure 3:**
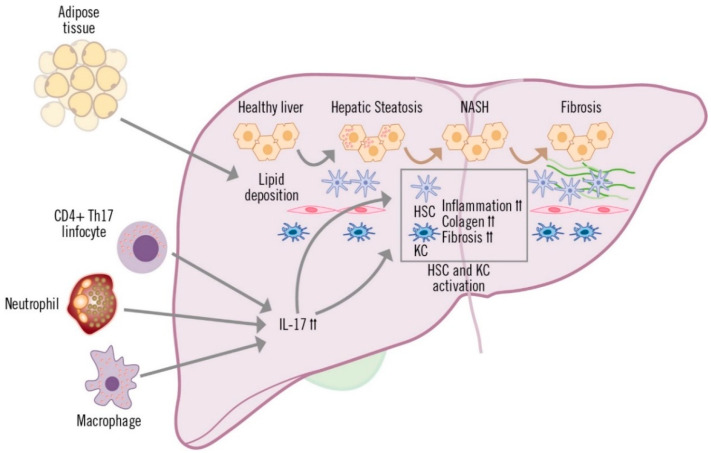
Role of IL-17 in the development of MAFLD.

The increased production of IL-17, whenever there is liver inflammation, is mainly due to CD4+ T (TH17) and CD8+ T cells (Tc17), although other innate immune cells, such as macrophages, NK cells, neutrophils and Tγδ cells are also capable of producing this cytokine [32,53,54]. The involvement of TH17 lymphocytes, macrophages and neutrophils has been described in the inflammatory process of MAFLD, which is generally accompanied by perivenular and periportal infiltration of these cells [53] (Figure 3). Various preclinical and clinical studies have shown that IL-17 produced by TH17 cells is implicated in multiple inflammatory processes in the liver [53].

IL-17 affects liver-resident cells in diverse ways. In hepatocytes, IL-17 mediates systemic inflammation and recruitment of inflammatory cells to the liver, and is also involved in fibrosis and insulin resistance [32,55,56]. In cholangiocytes, IL-17 promotes the uptake and differentiation of TH17 cells in the bile ducts, contributing to fibrosis and damage to the ducts [57]. Hepatic sinusoidal endothelial cells (HESC) participate in the exchange of mediators between the hepatic sinusoid and hepatocytes (space of Disse). Depending on the stimulation received, HESCs can inhibit cytokine secretion by TH17 and Th1 cells, or they can contribute to recruitment and migration, increasing adhesion of TH17 and Tc17 cells [58,59]. Hepatic stellate cells (HSC) and hepatic macrophages (or Kupffer cells; KC) are in the space of Disse, which is in contact with the hepatocytes and adjacent to the HESCs. IL-17 stimulates and is involved in the activation of HSCs, which respond by increasing expression of IL-17, pro-inflammatory cytokines (IL-6, IL-1β and TNF-α) and profibrogenic (TGF-β and alpha-smooth muscle actin protein [α-SMA]) receptors. HSC activation is amplified by the cooperation between IL-17 and TGF-β cytokines, leading to increased collagen production and the development of fibrosis [60,61,62]. Kupffer cells are activated by IL-17, due to surface expression of IL-17 receptor A and IL-17 receptor C and, similar to HSCs, they secrete pro-inflammatory mediators and the profibrogenic cytokine TGF-β, which increases activation of HSCs, further contributing to the progression of liver inflammation and fibrosis [54,61].

For better understanding of the effects of IL-17 in MAFLD, animal models of liver disease have been used to emulate the cascade of events that occur in the progression of MAFLD. Numerous studies have used a high-fat diet (HFD) to induce MAFLD and NASH (without progression to fibrosis) in mice, in order to identify the axis between TH17/regulatory T cells (Treg) and IL-17, considered to be an important part of the inflammatory factors underlying the transition from hepatic steatosis to NASH [34,63,64,65,66]. Administration of anti-IL-17 antibodies has been shown to improve liver function tests, inhibit KC activation and decrease levels of pro-inflammatory cytokines associated with inhibition of the NF-κβ pathway [67].

Several studies have used a choline-deficient diet in mice to induce NASH and fibrosis, demonstrating that infiltration of TH17 cells in the liver is a critical step in the initiation of NASH and in the development of fibrosis [68,69,70]. Carbon tetrachloride has been used to induce liver damage leading to liver fibrosis. In this stage of the disease, there is an imbalance between Treg and TH17 cells, which increases the number of TH17 cells, thereby stimulating the production of IL-17. In this model of liver injury, IL-17 orchestrates multiple mechanisms of profibrogenic action, facilitating the activation of HSCs and increasing production of IL-6, IL-1β, TNF-α, α-SMA and TGF-β [60,61].

Orthotopic tumor models in animals, and studies combining genotoxic agents with high-fat or choline-deficient diets, have demonstrated the key role of immune cells in progression from advanced stages of MAFLD (NASH and fibrosis) to HCC [66,71]. IL-17 is implicated in the progression of tumor cells in HCC through activation of the AKT signaling pathway [72]. Combination therapy with an anti-IL-17 monoclonal antibody (secukinumab), plus sorafenib, has been shown to better inhibit tumor growth and metastasis than sorafenib monotherapy [71]. Table 1 summarizes the results of various studies that have investigated the effects of anti-IL-17 antibodies in animal models.

Studies conducted in humans with chronic liver diseases have shown an increased intrahepatic infiltration of IL-17-secreting cells. A prospective study of 112 patients with MAFLD found that progression to NASH was correlated with the intrahepatic increase in TH17 lymphocytes, and a significant increase in the TH17/Treg ratio in peripheral blood [73]. Studies in patients with cirrhosis have found a significant increase in the percentage of TH17 cells present in peripheral blood mononuclear cells, and higher serum IL-17 levels, compared to healthy controls. IL-17 has also been shown to play an active role in the progression to HCC [66,74,75,76]. Liver inflammation is a critical component of tumor progression and IL-17 mediates neutrophil recruitment in the peritumoral stroma of HCC tissue [74].

In this clinical setting, several clinical trials have shown that the biologic agent secukinumab—an IL-17A inhibitor—can stabilize, or even achieve, a sustained improvement in metabolic and liver parameters. In those trials, aspartate aminotransferase (AST) and alanine transaminase (ALT) levels remained stable over the 52-week treatment period with secukinumab. By contrast, treatment with etanercept increased liver transaminase levels, a finding that is consistent with previous studies of anti-TNFα agents [77]. A recently published open-label, controlled clinical trial involving 130 patients with psoriasis and MetS, who were considered candidates for MTX or secukinumab, demonstrated that treatment with MTX increased liver enzymes while secukinumab had a neutral effect [78].

Other studies have found that significant increases in the number of TH17 cells in patients with HCC is correlated with tumor size, leading some authors to suggest that the percentage of TH17 cells and/or the TH17/Treg ratio could be useful prognostic markers in HCC [75,76].

## 4. Discussion

Given the close association between psoriasis and MAFLD, and the inflammatory factors common to both conditions, the need for close collaboration (both clinical and research) between dermatologists and hepatologists has become increasingly clear in recent years for best management of moderate-to-severe psoriasis and its comorbidities.

IL-17 is a pro-inflammatory molecule that enhances and perpetuates multiple inflammatory circuits relevant to both innate and adaptive immunity. As a result, this cytokine plays an important role in maintaining low-grade inflammation in several different organs and systems. Given that IL-17 plays a key role in both psoriasis and liver disease, it is important to determine how persistent inflammation in both organs (liver and skin) can promote, in psoriatic patients, a more rapid progression to steatohepatitis or cirrhosis, or even to hepatocellular carcinoma. Similarly, it would be of value to confirm how systemic inhibition of these inflammatory processes might affect not only the psoriasis manifestations, but also the evolution of MAFLD.

Currently, there are no approved pharmacotherapies for MAFLD. It has been suggested that inhibiting IL-17 might be a useful strategy for the management of the inflammation associated with both psoriasis and MAFLD conditions [22,65,77,78]. In this sense, there is strong evidence arising from recent animal studies, which suggests that IL-17 is involved in the progression of hepatic steatosis to MAFLD. Based on these findings, there is optimism in that the use of anti-IL-17 monoclonal antibodies, such as secukinumab, could prove beneficial for treating patients with psoriasis and MAFLD [79,80].

There is nevertheless a clear need for diagnosis protocols, and treatment/diagnostic algorithms that allow for early and global management of these patients. In addition, it would be desirable to create mechanisms to promote the exchange of information and facilitate the flow of patients between dermatology and hepatology specialties [22]. Consequently, a multidisciplinary approach to these conditions and related comorbidities (e.g., obesity and cardiovascular disease) is essential. In this line, experts from the European and American academies of dermatology, cognizant of the impact of MAFLD on patients with psoriasis, have formulated recommendations for the screening and management of relevant psoriasis comorbidities, including MetS and MAFLD [81,82]. Considering the most recent evidence to date, these professional academies consider that MAFLD should be screened for in patients with suggestive risk phenotypes, such as moderate-to-severe psoriasis and metabolic risk factors. These guidelines suggest that transaminase levels and an ultrasound should be included as part of an initial workup in this patient group. In addition, they propose an algorithm to monitor and follow up on these patients, which includes referring patients to a hepatologist if there are reasons to suspect that a patient may have liver involvement or disease. Moreover, they recommend that physicians consider the presence or absence of MAFLD when selecting psoriasis treatment. However, these recommendations do not seem to be universally implemented in most dermatologists’ clinical practices, nor has a specific guidance, protocol or circuit been established in most hospitals that facilitate referral of patients with concomitant psoriasis and MAFLD to the hepatology clinic for an appropriate evaluation.

In short, psoriasis and MAFLD seem to share a common pathogenetic mechanism in which IL-17 may play an essential role. Furthermore, psoriasis and MAFLD share the same molecular and immunological mechanisms as patients with MetS. A better understanding of the main factors and mechanisms involved in the pathogenesis of overlapping chronic inflammatory processes, such as psoriasis and MAFLD, could facilitate the development of more efficient therapeutic approaches for each of these conditions and potential treatment strategies with a shared benefit. Further consideration of anti-IL-17 antibodies through clinical trials is warranted to assess if this treatment strategy can benefit patients with concomitant MAFLD and psoriasis.

## Figures and Tables

**Table 1:** Impact of IL-17 inhibition in animal models. HFD: high-fat diet; MAFLD, metabolic-associated fatty liver disease; HCC: hepatocellular carcinoma; IL: interleukin; NASH: non-alcoholic steatohepatitis; LPS: lipopolysaccharide.

Model	Phase MAFLD	Intervention	Findings	Reference
Murine HFD + LPS	NASH	Anti-IL-17	decreased inflammatory cell infiltrates reduction of transaminase levels improved liver function	[34]
Murine HFD	NASH	Anti-IL-17	improved liver function inhibition of Kupffer cell activation reduction of pro-inflammatory cytokines	[67]
Murine	Fibrosis	Anti-IL-17	reduced liver damage	[65]
Murine	NASH HCC	Anti-IL-17	inhibition of disease development	[66]
Murine	HCC	Anti-IL-17 + sorafenib	inhibition of tumor growth and metastasis	[71]

## Data Availability

Not applicable.
